# Complete autonomic blockade reveals nitric oxide contribution to blood pressure regulation in obese Black women

**DOI:** 10.1007/s10286-024-01050-3

**Published:** 2024-08-01

**Authors:** Sharla Rahman, Alfredo Gamboa, Mohammad Saleem, Surat Kulapatana, André Diedrich, Italo Biaggioni, Annet Kirabo, Cyndya A. Shibao

**Affiliations:** 1 Department of Medicine, Epidemiology, Vanderbilt University School of Medicine, Nashville, TN 37212-8802 USA; 2 Department of Medicine, Division of Clinical Pharmacology, Vanderbilt University Medical Center, Room 536 Robinson Research Building, Nashville, TN 37212-8802 USA; 3 Department of Physiology, Faculty of Medicine, Siriraj Hospital, Mahidol University, Bangkok, 10700 Thailand; 4 Division of Clinical Pharmacology, Department of Medicine, Vanderbilt University Medical Center, P415C Medical Research Building IV, 2215 Garland Avenue, Nashville, TN 37232 USA

**Keywords:** Nitric oxide, Blood pressure, Autonomic blockade, Trimethaphan, L-NMMA, Obesity

## Abstract

**Purpose:**

Hypertension is one of the major causes of cardiovascular morbidity and mortality in the USA and disproportionately affects Black women. Endothelial-derived nitric oxide (eNO) substantially regulates blood pressure in humans, and impaired NO-mediated vasodilation has been reported in the Black population. Previous studies using an NO synthase inhibitor, N^G^-monomethyl-L-arginine (L-NMMA) did not fully determine the NO contribution to blood pressure because of baroreflex buffering. Therefore, in the present study we used trimethaphan, a ganglionic blocker, to inhibit baroreflex buffering and study NO modulation of blood pressure in Black women during L-NMMA infusion.

**Methods:**

L-NMMA at doses of 250 μg/kg per minute was infused in combination with trimethaphan at doses of 4 mg/min to eliminate baroreflex mechanisms. Heart rate (HR) was obtained with continuous electrocardiogram monitoring, and continuous blood pressure was measured with the volume clamp method. The increase in systolic blood pressure (SBP) during both infusions was used to estimate the contribution of NO to blood pressure.

**Results:**

Ten Black (age range 30–50 years, body mass index [BMI] 30–45 kg/m^2^), and nine White women (age range 30–50 years, body mass index 30–45 kg/m^2^) were enrolled in this study. During autonomic blockade, there was no difference in the decrease in SBP between Black and White women (− 20 ± 16.45 vs. − 24 ± 15.49 mm Hg, respectively; *P* = 0.659). When autonomic blockade was combined with L-NMMA, Black women had a significant increase in SBP compared to White women (54 ± 13.62 vs. 39 ± 09.64 mm Hg, respectively; *P* = 0.022, respectively).

**Conclusion:**

Autonomic blood pressure regulation was similar between Black and White women. However, NO contribution to blood pressure was significantly greater in Black women compared to White women.

**Registration:**

ClinicalTrials.gov: NCT01122407.

## Introduction

Obesity affects more than 30% of the US population and contributes to cardiovascular mortality, mostly due to diabetes and increased cardiovascular risks [1]. Black women are disproportionately affected by obesity compared with men or White women [2, 3]. Obesity precedes the development of hypertension and insulin resistance [4], both of which contribute to cardiovascular morbidity [5, 6]. The reported annual mortality rate among Black women due to cardiovascular events is 286 per 100,000 Black women [7]. Importantly, hypertension affects Black women at a younger age and is associated with a fivefold increase in coronary heart disease [8].

Obesity is associated with increased sympathetic activity. Microneurography, which directly measures baroreflex-modulated sympathetic traffic to skeletal muscle, is directly associated with body mass index (BMI) and fat mass [9–11]. We previously reported that this sympathetic overactivity contributes to obesity-associated hypertension in White women but not in Black women. Hence, other mechanisms may contribute to the pathophysiology of blood pressure regulation in this latter group.

Nitric oxide (NO) plays an important role in blood pressure regulation through its vasodilatory properties [12] and is arguably one of the most important metabolic modulators of blood pressure and vascular tone [13]. This endogenous-formed gas is synthesized in nearly all cell types, tissues, and organs in the human body [14] through three different isoforms of NO synthase enzyme, each with unique functional properties [15]. Endothelial-derived nitric oxide synthase (eNOS) is expressed in endothelial cells, platelets, the human placenta, and myocardial cells [14] and causes vasodilation with a subsequent decrease in blood pressure.

The effect of race on NO-mediated vasodilation has been assessed through the evaluation of vascular reactivity in response to the release of several factors, including NO, endothelium-derived hyperpolarization factor (EDHF), and prostaglandins; taken together, these studies reported an impaired vasodilatory response [16, 21]. Additional studies have also found a blunted endothelium-dependent vasodilation in response to a number of agonists, including acetylcholine [22, 23], methacholine [22, 24, 25], bradykinin [22], isoproterenol [25–27], sodium nitroprusside [22], exercise [28], mental stress [29], and, in some studies, also to ischemic stimulus on flow-mediated dilatation [28, 30, 31]. Despite the use of different methodologies and the limitations inherent to these techniques, there is consensus among the authors of these studies that endothelial-dependent vasodilation was abnormal in Blacks.

Importantly, the relative contribution of endothelial-derived NO (eNO) to blood pressure regulation in Black obese women is unknown. Therefore, the purpose of this study was to determine the contribution of NO on blood pressure regulation in Black obese women compared with White obese women. Considering that Black Americans have impaired endothelial-mediated vasodilation, we hypothesized that Black women would have less tonic restraint of blood pressure by NO compared with White women [32].

## Methods

### Study subjects

Subjects were recruited from the Vanderbilt University General Clinical Research Center volunteer database. Eligibility criteria included obesity, defined as BMI between 30 and 45 kg/m^2^, age range between 30 and 50 years, and Black and White women based on both parents being of the same race. Women who were pregnant or breastfeeding and had a history of coronary artery disease, heart failure, renal or liver failure, and significant weight gain or loss in the past 3 months were excluded. The study was approved by the Vanderbilt University Institutional Review Board, and all participants gave written informed consent before study entry. The study was registered in clinicaltrials.gov (NCT01122407). The data reporting the decrease in blood pressure with trimethaphan (TMT) was previously published as part of a larger cohort [33]; the N^G^-monomethyl-l-arginine (L-NMMA) data have not been previously published.

### Experimental design

All subjects underwent a thorough clinical examination and laboratory analyses (cell blood count, basal metabolic panel, urinalyses, and urinary pregnancy test) prior to enrollment. Subjects were given a weight-maintenance sodium-balanced diet (50% carbohydrates, 30% fat, 20% protein, 150 mEq sodium) 3 days prior to the infusion protocol. They were also told to refrain from all food and beverages containing methylxanthines. The study was conducted in the morning, after a 12-h overnight fast.

**Fig. 1:**
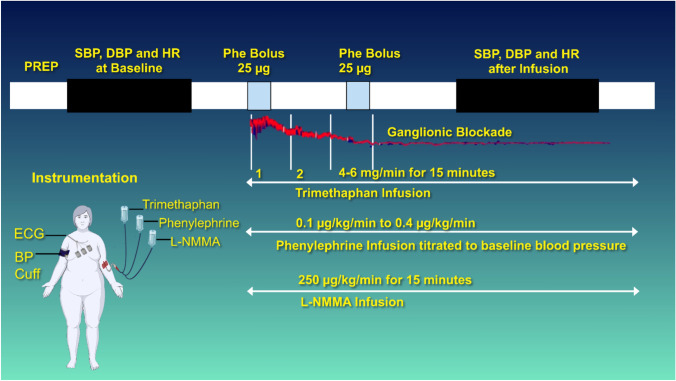
Pictorial depiction of the experiment design. Blood pressure and R-R intervals (red) were measured during ganglionic blockage using trimethaphan infusion with escalating dosages (1, 2, 4, up to 6 mg/min). Trimethaphan infusion was maintained after the establishment of the complete blockade. Baseline BP was restored by titrated Phe infusion. NO was blocked by L-NMMA infusion at 250 µg/kg/min.* BP* Blood pressure, *DBP* diastolic BP, *ECG* electrocardiogram, *HR* heart rate, *L-NMMA* N^G^-monomethyl-L-arginine,* NO* nitric oxide, *Phe* phenylephrine, *SBP* systolic BP

The infusion protocol is presented in Fig. 1. Subjects were fitted with two intravenous (IV) catheters, one each in the antecubital vein of each arm. One arm had three infusion ports connected to the IV catheter for TMT (Cambridge Pharmaceuticals, Newcastle upon Tyne, UK), phenylephrine (an alpha-1-adrenergic agonist), and L-NMMA infusion. The opposite arm was used for blood draws and phenylephrine boluses. Heart rate (HR) was obtained with continuous electrocardiogram monitoring, and continuous blood pressure was measured with the volume clamp method (Finapres, Enschede, The Netherlands) and intermittently with automated brachial cuff pressure with standard sphygmomanometry. Cardiac output (CO) was measured at intervals with the inert gas re-breathing method (Innocor CO; COSMED Nordic ApS, Odense, Denmark) [34].

All subjects were allowed to rest in a quiet, thermoneutral environment for 30 min after instrumentation. Baseline hemodynamic parameters were recorded. Phenylephrine boluses were used to confirm abolished baroreflex-mediated prolongation of R-R intervals.

TMT was administered for 15 min, and phenylephrine boluses were repeated, starting at a dose of 2.5 ug until an increase of 25 mm Hg was achieved in systolic blood pressure (SBP) [35, 36]. Autonomic withdrawal can cause a mild decrease in blood pressure due to the low sympathetic tone present in the supine resting position. Therefore, we infused phenylephrine starting at a rate of 0.01 ug/kg/min and titrated to restore blood pressure to baseline levels; the infusion was maintained constant for the duration of the study. Then, L-NMMA was infused at 250 ug/kg per minute for 15 min or until SBP reached 150 mm Hg.

### Estimation of autonomic blockade using spectral analysis of blood pressure and heart rate

All the of physiological data were recorded through a WINDAQ data acquisition system. The recordings were digitized with 14-bit resolution and 500-Hz sample frequency. An offline custom-written software program for data analysis in PV-Wave language (PV-wave; Visual Numerics Inc., Houston, TX, USA) was used to analyze data (DIANA, AD, Vanderbilt University, Nashville, TN, USA). A QRS detection algorithm, modified from Pan and Tompkins [37], generated beat-to-beat values. Beat-to-beat values of the RR interval and blood pressure were interposed, low pass filtered (cutoff: 2 Hz), and resampled (4 Hz).

Data segments of interest were used for the spectral analysis. Linear trends were removed, and power spectral density was estimated with a Fast Fourier Transform (FFT)-based algorithm using Welch’s method, using at least three segments of 256 data points with 50% overlapping and a Hanning window. According to task force recommendations, the power in the frequency ranges for very low frequencies (0.003 to < 0.04 Hz), low frequencies (LF: 0.04 to < 0.15 Hz), and high frequencies (HF: 0.15 to < 0.40 Hz) were calculated for each interval [38].

### Spectral baroreflex transfer function gain

Bivariate power spectral analysis provided useful information about the temporal fluctuations between different hemodynamic parameters, such as HR and blood pressure. We estimated the power spectra, cross spectra, phase, coherence, and transfer function gain of SBP and R-R interval time series using FFT with a segment length of 256 s resampled with 4 Hz. The baroreflex gain was determined as the mean magnitude value of the transfer function in the low-frequency band with a negative phase and squared coherence value > 0.5.

#### Endpoints

The primary endpoint was the increase in SBP during combined NO synthase inhibition and autonomic blockade.

Secondary endpoints included the change in DBP and HR during combined NO synthase inhibition and autonomic blockade. Other surrogate measurements of sympathetic activity (low-frequency variability of SBP [LF_SBP_]), parasympathetic activity (high-frequency variability of HR [HF_RRI_]), and spontaneous baroreflex sensitivity (BRS) were also measured.

## Statistical analysis

Standard graphing and screening techniques were employed to detect outliers and to ensure data accuracy. The data were assessed for normality by the Anderson–Darling test, Shapiro–Wilk test, and Kolmogorov–Smirnov test; all three tests were used to check the normality. If normality was violated, we applied a non-parametric method of analysis. Demographic data were presented as means with standard errors of the mean (SEM). We used a parametric Welch’s Correction test to compare the differences in primary outcomes (Delta SBP) and secondary outcomes as previously defined. All tests were two-tailed, and a *P* value of < 0.05 was considered to be significant. Analyses were performed using GraphPad Prism statistical software (version 9.5.0; GraphPad Software, San Diego, CA, USA).

## Results

### Demographic characteristics

A total of 19 (10 Black and 9 White) premenopausal and obese women were enrolled in the study. The enrolled subjects were mostly hypertensive (14 hypertensive and 4 healthy women). Demographic characteristics are shown in Table 1. Total cholesterol levels were lower in the Black women than in the White women (mean ± SD, 141 ± 52.91 vs. 187 ± 25.29 mg/dL, *P* = 0.051). No significant differences were found in SBP while resting and while upright between the groups. There were no significant differences found in urine analysis of sodium and creatinine levels between Black and White women (139 ± 1.94 vs. 138 ± 1.27 mEq/L, respectively, *P* = 0.251; 0.81 ± 0.11 vs. 0.71 ± 0.11 mg/dL, respectively, *P* = 0.070).

**Table 1:** Demographics and baseline characteristics of subjects enrolled in the study

Parameters	Black women (*N* = 10)	White women (*N* = 9)	*P*-value^a^
Age (years)	39 ± 6.43	42 ± 8.21	0.438
Height (cm)	164 ± 3.66	162 ± 6.47	0.420
Weight (kg)	93 ± 10.97	92 ± 14.03	0.719
BMI (kg/m^2^)	34 ± 3.99	35 ± 4.04	0.837
SBP supine (mmHg)	122 ± 12.22	119 ± 13.30	0.607
SBP seated (mmHg)	126 ± 13.86	125 ± 7.07	0.830
SBP standing (mmHg)	130 ± 14.94	123 ± 20.09	0.421
DBP supine (mmHg)	78 ± 7.56	72 ± 11.84	0.224
DBP seated (mmHg)	79 ± 8.69	76 ± 8.20	0.428
DBP standing (mmHg)	87 ± 11.57	80 ± 9.68	0.174
HR supine (bpm)	64 ± 11.40	68 ± 9.06	0.468
HR seated (bpm)	72 ± 10.02	73 ± 8.60	0.853
HR standing (bpm)	77 ± 10.58	80 ± 10.62	0.634
MAP supine (mmHg)	93 ± 8.71	88 ± 11.79	0.319
Glucose (mg/dL)	91 ± 4.76	85 ± 7.87	0.082
Insulin (mcU/mL)	7 ± 3.36	5 ± 3.22	0.447
Sodium (mEq/L)	139 ± 1.94	138 ± 1.27	0.251
Potassium (mEq/L)	04 ± 0.39	04 ± 0.48	0.909
Creatinine (mg/dL)	0.81 ± 0.11	0.71 ± 0.11	0.070
LDL cholesterol (mg/dL)	91 ± 20.71	106 ± 19.35	0.192
HDL cholesterol (mg/dL)	53 ± 7.81	57 ± 6.44	0.367
Total cholesterol (mg/dL)	141 ± 52.91	187 ± 25.29	0.051
Triglycerides (mg/dL)	60 ± 31.28	117 ± 59.99	0.103
Total body fat free mass (kg)	53 ± 5.02	49 ± 5.85	0.204
Total body fat mass (kg)	39 ± 7.52	42 ± 9.71	0.416
Waist circumference (cm)	102 ± 4.91	98 ± 7.29	0.246

Values in table are presented as the mean ± standard deviation

*BMI* Body mass index, *DBP* diastolic blood pressure, *HDL* high-density lipoprotein, *HR* heart rate, *LDL* low-density lipoprotein, *MAP* mean arterial pressure,*NO* nitric oxide, *SBP* systolic blood pressure

^a^*P* values are for the differences between Black and White women, by Welch’s Correction test

### Effect of autonomic blockade on cardiovascular parameters

**Fig. 2:**
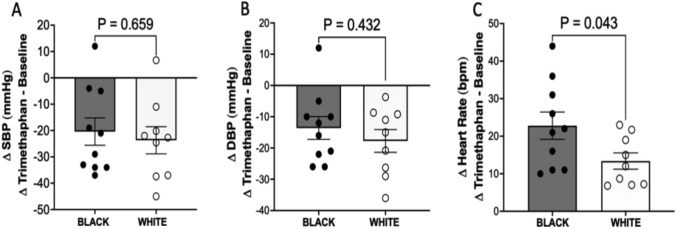
Effect of autonomic withdrawal induced by the ganglionic blockade on blood pressure and heart rate in Black and White women. **a** Delta values of SBP at the end of trimethaphan infusion and changes in SBP induced by the autonomic blockade. **b** Delta values of DBP after the treatment with trimethaphan and resulting changes in DBP. **c** Delta heart rate values after trimethaphan infusion and any changes in heart rate induced by an autonomic blockade. Data are presented as mean ± standard error of the mean. Statistical analysis was performed using Welch’s Correction test. *N* = 10 for Black women and *N* = 9 for White women. *P* < 0.05 was set for statistical significance. DBP Diastolic blood pressure, SBP systolic blood pressure

There was no difference between Black and White women in the decrease in SBP (− 20 ± 16.45 vs. − 24 ± 15.49 mmHg, respectively, *P* = 0.659; Fig. 2a) and DBP (-14 ± 11.53 vs. − 18 ± 10.88 mm Hg, respectively; *P* = 0.431; Fig. 2b). HR significantly increased more in Black women than in White women (23 ± 11.52 vs. 13 ± 16.54 bpm, respectively, *P* = 0.043; Fig. 2c).

There were no significant differences between Black and White women in CO (mean ± SEM, − 1 ± 01.13 vs. − 1 ± 01.07 L/min, respectively, *P* = 0.446) and systemic vascular resistance (SVR; − 1 ± 01.78 vs. − 1 ± 01.14 dynes/s/cm^−5^, respectively, *P* = 0.786)). There was also no difference in the decrease in stroke volume (SV) between the groups (− 30 ± 12.26 vs. − 30 ± 24.57 mL/beat, *P* = 0.978). The changes in CO, SV, and SVR before and after autonomic blockade are shown in Table 2.

**Table 2:** Changes in cardiac output, stroke volume, and systemic vascular resistance before and after autonomic blockade in Black and White

Experimental condition	Cardiac output		Stroke volume				Systematic vascular resistance		
	Black women	White women	*P*-value^a^	Black women	White women	*P*-value^a^	Black women	White women	*P*-value^a^
Baseline	6 ± 0.35	8 ± 0.69	0.041	92 ± 6.42	111 ± 9.07	0.139	15 ± 0.95	11 ± 0.75	0.011
TMT	5 ± 0.54	7 ± 0.39	0.079	62 ± 8.39	82 ± 4.21	0.148	15 ± 2.15	9 ± 0.32	0.034
L-NMMA	6 ± 0.49	7 ± 0.15	0.333	80 ± 9.34	86 ± 4.36	0.630	20 ± 1.71	16 ± 0.31	0.073

Values are presented in table as the mean ± standard error of the mean (SEM)

*L-NMMA* N^G^-monomethyl-L-arginine, *TMT* trimethaphan

^a^ *P* values are for the differences between Black and White women, by the Mann–Whitney test

Baseline HF_RRI_ values were similar between the groups (mean ± SEM, 745 ± 215.90 vs. 845 ± 655.90 ms^2^, *P* = 0.114), as were baseline LF_RRI_ values (915 ± 273.80 vs. 792 ± 493.20 ms^2^, *P* = 0.167) (Table 3). During the ganglionic blockade, the difference in HF_RRI_ values was significant between Black and White women (2 ± 0.45 vs. 24 ± 19.01 ms^2^, respectively *P* = 0.029), while LF_RRI_ in Black and White women decreased to similar values (7 ± 3.50 vs. 30 ± 25.07 ms^2^, respectively, *P* = 0.662). The result shows a significant difference in HF_RRI_ HR variability in both groups. The ratio between low- and high-frequency HR variability (LF_RRI_/HF_RRI_) was higher in Black women than in White women during the ganglionic blockade (4 ± 7.91 vs. 1 ± 1.32, respectively, *P* = 0.446).

**Table 3:** Changes in spectra analysis parameters induced by autonomic blockade

Parameters	Baseline			Ganglionic blockade		
	Black women	White women	*P*-value^a^	Black women	White women	*P-*value^a^
HF_RRI_ (ms^2^)	745 ± 215.90	845 ± 655.90	0.114	2 ± 0.45	24 ± 19.01	0.029
LF_RRI_ (ms^2^)	915 ± 273.80	792 ± 493.90	0.167	7 ± 3.56	30 ± 25.07	0.662
SD_RRI_ (ms)	64 ± 7.35	47 ± 10.57	0.219	9 ± 2.70	13 ± 3.28	0.424
RMSSD (ms)	67 ± 9.09	39 ± 13.33	0.104	3 ± 0.53	6 ± 2.10	0.206
LF_RRI_/HF_RRI_	1 ± 1.27	1 ± 0.75	0.995	4 ± 7.91	1 ± 1.32	0.446
BRS (ms/mmHg)	9 ± 0.94	8 ± 2.21	0.236	1 ± 0.42	3 ± 1.23	0.081
LF_SBP_ (mmHg^2^)	10 ± 1.94	8 ± 2.04	0.541	3 ± 1.51	1 ± 0.29	0.867

Values in table are presented as the mean ± standard error of the mean (SEM)

*BRS* Baroreflex sensitivity,* HF*_*RRI*_ high-frequency variability of heart rate, * LF*_*SBP*_ low-frequency variability systolic blood pressure, * LF*_*RRI*_ low-frequency variability of heart rate, *RMSSD* square root of mean squared successive differences,* SD*_*RRI*_ standard deviation of the R–R interval

^a^*P* values are for the differences between Black and White women, by the Mann–Whitney test. *P* values for LF_RRI_/HF_RRI_ are reported using the one-way analysis of variance (ANOVA) test

There was no significant difference in the change in the values of the blood pressure variability parameters when compared at baseline and after autonomic blockade between the groups. LF_SBP_ values at baseline were not significantly different between Black and White women (mean ± SEM, 10 ± 1.94 vs. 8 ± 2.04 mm Hg^2^, respectively, *P* = 0.541) (Table 3). LF_SBP_ values decreased after the ganglionic blockade to the same values in both groups (3 ± 1.51 vs. 1 ± 0.29 mm Hg^2^; *P* = 0.867). There was no difference in BRS between Black and White women at baseline (9 ± 0.94 vs. 8 ± 2.21 ms/mmHg, *P* = 0.236). After the autonomic blockade, the baroreflex function decreased in both groups to the same levels (1 ± 0.42 vs. 3 ± 1.23 ms/mm Hg, *P* = 0.081).

During autonomic blockade, the standard deviation heart rate variability (SD_RRI_) showed no significant difference in the decrease between Black and White women during blockade (mean ± SEM, 9 ± 02.70 vs. 13 ± 03.28 ms^2^, respectively, *P* = 0.424) (Table 3). There were no significant differences in the square root of mean squared successive differences (RMSSD) between the groups during blockade (3 ± 0.53 vs. 6 ± 02.10 ms, *P* = 0.206). Baroreflex function declined during TMT infusion from baseline values in both Black and White women (9 ± 0.93 to 1 ± 0.42 ms/mmHg [Black women], *P* = 0.001; 8 ± 2.21 to 3 ± 01.23 ms/mm Hg [White women], *P* = 0.011).

 During the ganglionic blockade, the HF_RRI_ and LF_RRI_ HR variability significantly decreased in both groups. The values at baseline and during autonomic blockade values were also compared in each group. In Black women, high- and low-frequency HR variability significantly decreased during the ganglionic blockade compared to baseline values (745 ± 215.90 to 2 ± 0.45 ms^2^, *P* = 0.001; 915 ± 273.80 to 7 ± 3.56 ms^2^, *P* = 0.001). In White women, HF_RRI_ and LF_RRI_ HR variability significantly decreased during the ganglionic blockade compared to baseline values (845 ± 655.9 to 24 ± 19.02 ms^2^, *P* = 0.001; 792 ± 493.90 to 30 ± 25.07 ms^2^, *P* = 0.001).

### Effect of the L-NMMA on SBP, DBP, and HR of black women compared to white

**Fig. 3:**
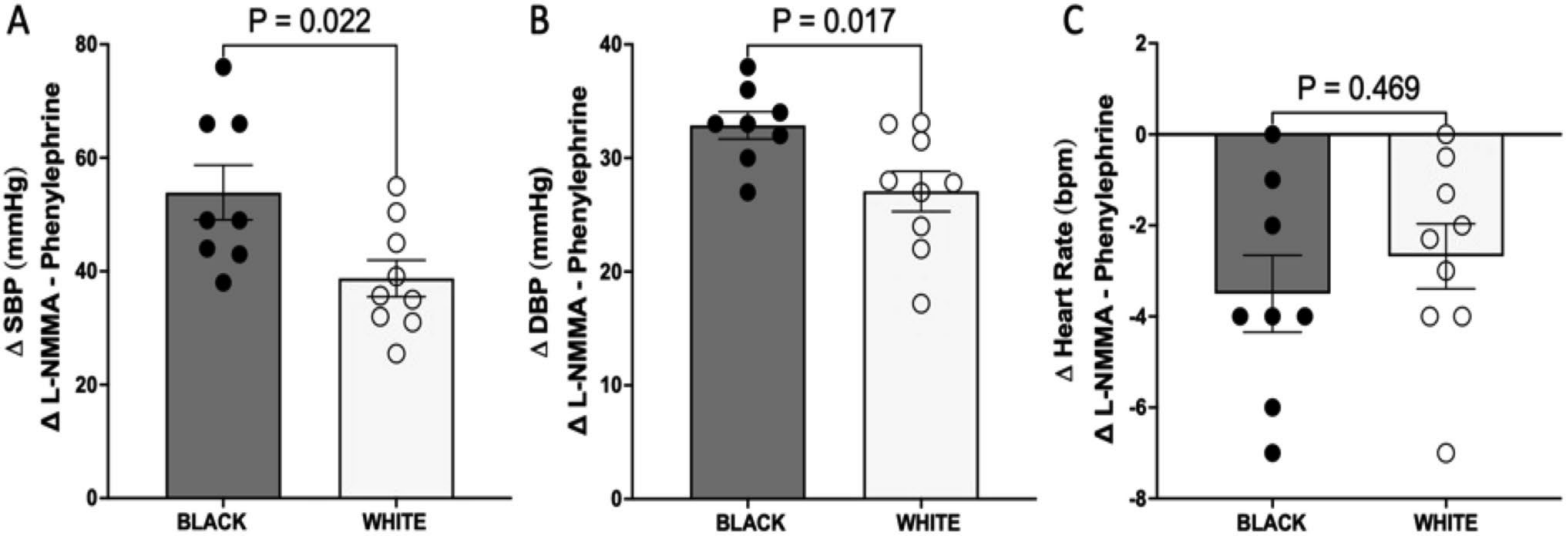
Effect of L-NMMA on blood pressure and heart rate in the absence of a functional autonomic nervous system in Black and White women, respectively. **a** Delta values of SBP after infusion of L-NMMA and changes in SBP induced by NOS inhibition. **b** Delta values of DBP after L-NMMA infusion and resulting changes in DBP. **c** Delta values of heart rate after L-NMMA infusion and changes in heart rate induced by NOS inhibition. Data are presented as mean ± standard error of the mean. Statistical analysis was performed using Welch’s Correction test. *N* = 10 for Black women and *N* = 9 for White women. *P* < 0.05 was set for statistical significance.* DBP* Diastolic blood pressure, *L-NMMA* N^G^-monomethyl-L-arginine,* NOS SBP* systolic blood pressure

In each group, the L-NMMA infusion at 250 μg/kg per minute increased SBP and DBP, with the increase significantly greater in Black women than in White women (54 ± 13.62 vs. 39 ± 09.64 mmHg, *P* = 0.022; 33 ± 03.39 vs. 27 ± 05.30 mmHg, *P* = 0.016; Fig. 3a, b, respectively). There was no significant change in HR in Black women compared to White women (− 4 ± 2.39 vs. − 3 ± 2.14 bpm, *P* = 0.469; Fig. 3c).

## Discussion

Our main finding was that during NO synthase inhibition in the context of autonomic blockade, the pressor response was more remarkable in Black women than in White women, indicating that endogenous endothelial derived NO significantly contributes to blood pressure modulation to a greater extent in Black women compared with White women.

A previous study [39], using intravenous infusion of L-NMMA doses ranging from 200 to 800 μg/kg per minute, found only a mild increase in blood pressure in healthy volunteers. The authors also reported a mild HR decrease, indicating active baroreflex buffering. To overcome this limitation, our group previously reported that the combined use of L-NMMA and TMT, a ganglionic blocker, helped to measure NO modulation of blood pressure without the confounding effect of baroreflex buffering. Subjects that participated in this study had a moderate increase in SBP with lower doses of L-NMMA [32]. Using this technique, we are currently reporting a similar increase in SBP in White women but a two-fold increase in blood pressure in Black women. Hence, this latter population depends on their endogenous eNO to maintain their blood pressure within normal limits.

Racial differences in NO-induced vasodilation in response to mental stress have been previously studied. Forearm blood flow measurements during intra-arterial infusion of L-NMMA and mental stress were suppressed in Black subjects but not in White subjects [29]. A similar study found attenuated NO-induced vasodilation in Black subjects during resting conditions [40]. Importantly, these studies evaluated NO-induced vasodilation in the microcirculation, which could play a role in the development of future hypertension in these healthy subjects; however, they do not directly measure the tonic contribution of NO to systemic hemodynamics.

In our study, both groups showed a similar decrease in blood pressure during TMT infusion, indicating that sympathetic vasoconstriction activity was equal between groups, as we previously reported in the literature [33]. Importantly, we found a robust increase in HR in Black women during TMT infusion compared with White women, which could indicate an increased sympathetic and cardiovagal regulation of HR. We argue that this difference in HR may not affect blood pressure directly, given that systemic hemodynamic values such as CO, SVR, and SV did not change with TMT.

While our study focused on the acute effects of NO synthase inhibition and autonomic blockade on blood pressure regulation in Black and White obese women, we did not assess for other mechanisms, such as the renin–angiotensin–aldosterone system (RAAS), that may contribute to the differences observed in blood pressure [41]. It is well-documented that Black individuals have higher plasma renin activity than White individuals. This could be, in part, due to the higher prevalence of salt sensitivity. Previous studies demonstrated that salt sensitivity is an independent risk factor for mortality and morbidity due to cardiovascular disease [42–44]. Our protocol partially controlled sodium intake by providing subjects with a salt-balanced diet for 3 days before the study day.

Another important consideration is the possibility of differential effects of TMT on the arterial (high-pressure) and venous (low-pressure) systems between Black and White individuals. While our study primarily focused on systemic hemodynamics, including blood pressure and HR, variations in the response of the venous system to autonomic manipulation could impact cardiovascular outcomes differently in different racial groups. Unleashed reflexes of the venous system, such as Bainbridge-like reflexes, triggered by blocking the dominant arterial baroreflexes, may lead to tachycardia as a compensatory response to increased venous return. These reflexes could potentially vary in sensitivity or magnitude between Black and White individuals, thereby influencing overall cardiovascular responses [45].

In conclusion, using a validated approach to determine the contribution of eNO on blood pressure by measuring the effect of systemic NOS inhibition during autonomic blockade, we found that eNO was the most important blood pressure modulator in Black obese women.

### Limitations

Our study had several limitations. First our sample size was small; however, we performed in-depth phenotyping of study participants using pharmacological probes that acutely block sympathetic, parasympathetic, and NO function, which controls for differences in these parameters among groups. The other limitation was the use of phenylephrine to restore the blood pressure to baseline level during TMT infusion and before the infusion of L-NMMA. It could be possible that the excessive increase in SBP in Black women was secondary to hypersensitivity to alpha-1 receptors. However, we used the same doses of phenylephrine in both groups to restore the SBP to baseline values, and the increase in SBP with phenylephrine bolus (data not shown) was similar in our Black and White subjects. Further, we enrolled obese women and, therefore, our findings may not extend to the larger population of obese women with cardiometabolic conditions or men.

Additionally, it is important to acknowledge the potential dominance of venous constriction effects of phenylephrine in our experimental protocol. Phenylephrine, an alpha-1 adrenergic agonist used to restore blood pressure to baseline levels during TMT infusion, primarily acts by constricting both arterial and venous vessels. In turn, low-dose alpha-activating drugs like phenylephrine may exert a more pronounced effect on venous constriction and venous return, which could have influenced our observed cardiovascular responses differently in Black and White women. This differential response to phenylephrine administration could potentially confound interpretations of autonomic function and blood pressure regulation [46].

By addressing these limitations, future research can further elucidate the complex interplay between autonomic regulation, vascular physiology, and racial disparities in cardiovascular health.

## Abbreviations

BRS: Baroreflex sensitivity
DBP: Diastolic blood pressure
EDHF: Endothelium-derived hyperpolarization factor
eNO: Endothelial-derived nitric oxide
eNOS: Endothelial nitric oxide synthase
HR: Heart rate
HF_RRI_: High-frequency variability of HR
LF_RRI_: Low-frequency variability of HR
LF_SBP_: Low-frequency variability of SBP
L-NMMA: N^G^-monomethyl-l-arginine
NO: Nitric oxide
RMSSD: Root mean square of successive differences in R-R intervals
SBP: Systolic blood pressure
SD_RRI_: Standard deviation of R-R intervals
TMT: Trimethaphan
